# Flower Colour Modification of Chrysanthemum by Suppression of *F3'H* and Overexpression of the Exogenous *Senecio cruentus F3'5'H* Gene

**DOI:** 10.1371/journal.pone.0074395

**Published:** 2013-11-08

**Authors:** Huang He, Hu Ke, Han Keting, Xiang Qiaoyan, Dai Silan

**Affiliations:** 1 College of Landscape Architecture, Beijing Forestry University, Beijing, China; 2 Key Laboratory of Plant Germplasm Enhancement and Speciality Agriculture, Wuhan Botanical Garden, Wuhan, Hubei, China; RIKEN PSC, Japan

## Abstract

Chrysanthemum (*Chrysanthemum × morifolium*) is one of the most important ornamental plants in the world. They are typically used as cut flowers or potted plants. Chrysanthemum can exhibit red, purple, pink, yellow and white flowers, but lack bright red and blue flowers. In this study, we identified two chrysanthemum cultivars, *C × morifolium* ‘LPi’ and *C × morifolium* ‘LPu’, that only accumulate flavonoids in their ligulate flowers. Next, we isolated seven anthocyanin biosynthesis genes, namely *CmCHS*, *CmF3H*, *CmF3*’H, CmDFR, *CmANS*, *CmCHI* and *Cm3GT* in these cultivars. RT-PCR and qRT-PCR analyses showed that CmF3′H was the most important enzyme required for cyanidin biosynthsis. To rebuild the delphinidin pathway, we downregulated *CmF3*’*H* using RNAi and overexpressed the *Senecio cruentus F3*′*5*′*H* (*PCFH*) gene in chrysanthemum. The resultant chrysanthemum demonstrated a significantly increased content of cyanidin and brighter red flower petals but did not accumulate delphinidin. These results indicated that *CmF3*′*H* in chrysanthemum is important for anthocyanin accumulation, and *Senecio cruentus* F3′5′H only exhibited F3′H activity in chrysanthemum but did not rebuild the delphinidin pathway to form blue flower chrysanthemum.

## Introduction

Flower colour is determined on the basis of flavonoids, carotenoids and betalains [1]. Anthocyanins, a group of secondary metabolites belonging to the flavonoid family, demonstrate a wide range of orange to red and purple to blue flowers, which can attract pollinators and, importantly, protect against damage from UV irradiation. In addition, anthocyanins performs as key signals between plants and microbes [2,3].

**Figure 1:**
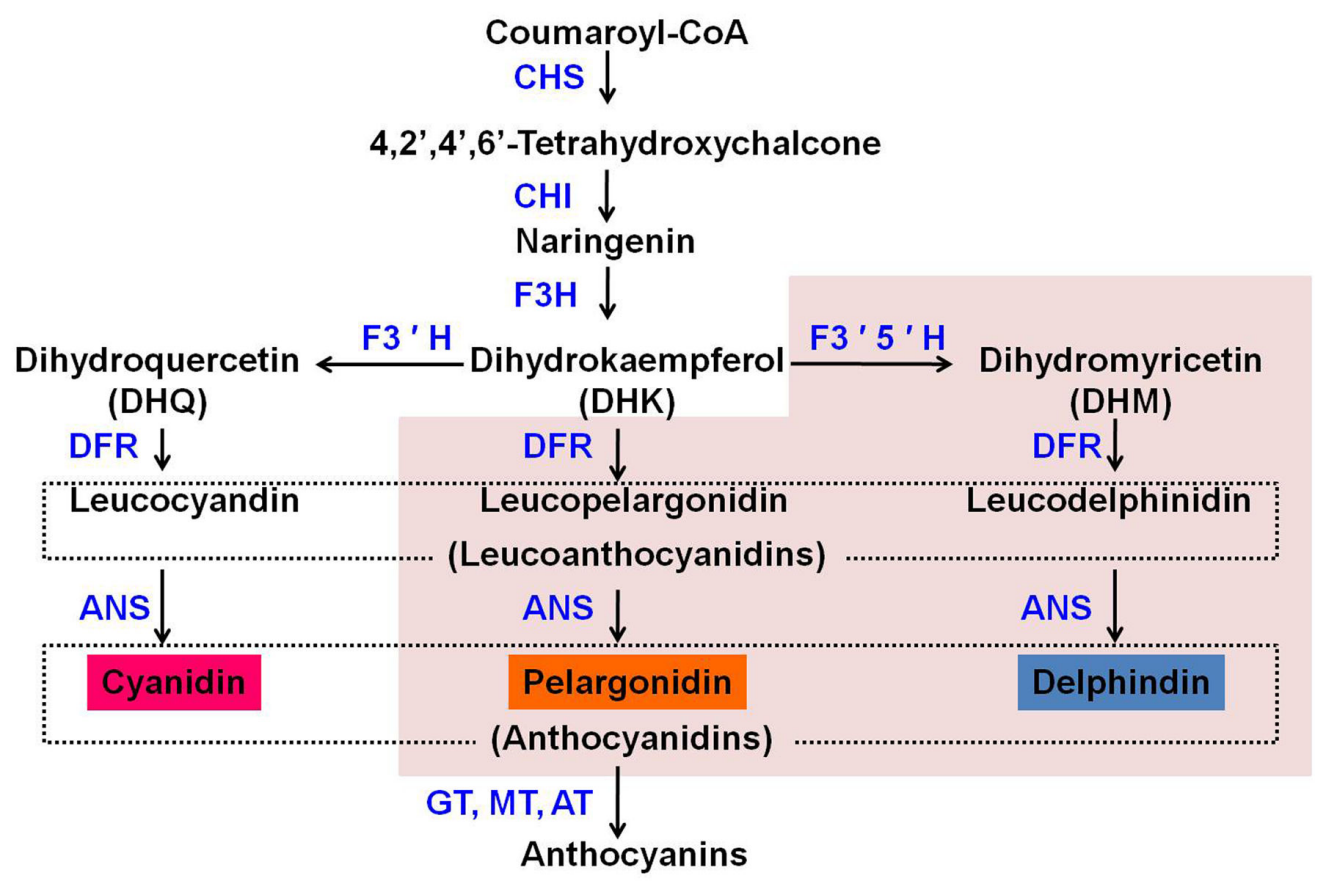
The flavonoid biosynthetic pathway leading to the synthesis of anthocyanins. The shaded area presents the inexistent pathway in chrysanthemum in this study.

The anthocyanin biosynthesis pathway (ABP) is a branch of the flavonoid biosynthesis pathway that is derived from the phenylpropanoid biosynthesis pathway. Briefly, the ABP begins with the formation of chalcones by the chalcone synthase enzyme (CHS). Next, chalcone isomerase (CHI) converts chalcone into naringenin [4]. Naringenin is then hydroxylated at the 3′ position of its central ring by flavanone 3-hydroxylase (F3H) to produce dihydrokaempferol (DHK). DHK can then be further hydroxylated at the 3′ position or at both the 3′ and 5′ positions of the B-ring to produce dihydroquercetin and dihydromyricetin, respectively [5] (Figure 1) DHK, dihydroquercetin, and dihydromyricetin generally result in the production of brick-red/orange pelargonidin-, red/pink cyanidin-, and blue/violet delphinidin-based pigments, respectively [5]. Thus, the establishment of these three biosynthesis pathways is essential for diverse flower colours. 

Each species usually accumulates limited kinds of anthocyanins and exhibits limited kinds of flower colour on the basis of the expression of a specific set of biosynthetic genes, the substrate specificity of key enzymes and the temporal and spatial regulation of the biosynthetic genes [6]. Thus, the colour range in many ornamental plants is limited by the genetic background of the species [7,8], and genetic modification (GM) is the only effective way to overcome this limitation [9] .Many ornamental plants lack cultivars of the violet to blue flower due to the absence of delphinidin-based anthocyanins. Thus, GM of blue coloured flowers is important to horticultural breeders [10]. There are two main strategies employed to modify flower colour using transgenic approaches. One is the control of endogenous flavonoid pigments in flowers both quantitatively and qualitatively, and the other strategy is to accumulate non-native pigments in flowers [11]. Both strategies are effective. In rose, to out compete the endogenous DFR for substrates dihydroquercetin, *RcDFR* was targeted for downregulation via RNAi-mediated silencing. The resulting transgenic plants, which also expressed pansy *F3*'*5*'*H* and Dutch iris *DFR* genes, produced flowers that exclusively accumulated delphinidin-based anthocyanins with a concomitant colour change towards blue[12]. In carnation, over-expression of a petunia *F3*'*5*'*H* gene in a pelargonidin producing carnation cultivar produces petals in which delphinidin derivatives contribute to about 70% of total anthocyanins[13]. In gentian, suppression of the *F3*'*5*'*H* gene decreased delphinidin derivatives and resulted in magenta flower colours, which could be utilised as elite sources to breed gentian plants in the near future [14]. In *Cyclamen persicum*, suppression of the endogenous *F3*'*5*'*H* transcript resulted in a hue shift from purple to red/pink in one cultivar [15] Of these examples, we concluded that the most promising strategy to obtain blue flowers was to suppress endogenous competition enzymes of the substrate for F3′5′H and to simultaneously introduce an appropriate exogenous *F3*′*5*′*H* gene as well as downstream biosynthesis genes of the ABP pathway.

Chrysanthemum (*Chrysanthemum* × *morifolium* Ramat.) is one of the most important ornamental plants in the world. The main anthocyanin biosynthesis pathway in chrysanthemum was the cyanin-based pathway [1], so there are no blue pigments in any of the chrysanthemum cultivars due to the lack of delphinidin-based anthocyanins and deficiency in F3′5′H [16–18]. Compared to rose and carnation, molecular breeding of blue colour flower in chrysanthemum is in the nascent stage, although molecular genetic technology has been widely used to improve other aspects of chrysanthemum cultivar [19]. To date, no studies focused on molecular breeding involving flavonoid pigments for chrysanthemum and no reports on the isolation and functional analysis of ABP genes of chrysanthmum. 

 In the present study, we isolated seven key structural genes involved in anthocyanin biosynthesis in chrysanthemum, and suppressed a key nodal gene *F3*′*H* in chrysanthemum to block cyanidin-based anthocyanin pathway. Simultaneously, the *F3*′*5*′*H* gene from *Senecio cruentus* (*PCFH*), which produces new delphinidin derivatives in the corollas of transgenic tobacco plants [20], was introduced into the chrysanthemum to create flower colour-modified chrysanthemums.

## Materials and Methods

### Plant materials

The *in vitro* plantlets of different coloured series (white, red, pink and purple) of *Chrysanthemum × morifolium* ‘Lijin’ were germinated at 22°C under illumination in a 14 h light period during vegetative growth and 8 h light period during reproductive growth with 40% relative humidity in an artificial climate chamber in Beijing Forestry University. The white, red, pink and purple cultivars were named LW, LR, LPi and LPu, respectively, in this study.

There are five stages in the development of the capitulum: S1, ray florets were not yet out of bract (0-5 mm in ray flower length); S2, ray flowers were acicular and had barely outgrew the bract (5 mm in ray flower length); S3, ray flowers clearly outgrew the bract, but the capitulum was compact (5-8 mm in ray flower length); S4, ray flowers were opening and the angle between the ray flowers and stem was over 90° (12-18 mm in ray flower length); and S5, fully opened flower and the angle between ray flowers and stem was nearly 90° (16-20 mm in ray flower length). The five different stages of ray flower development were frozen in liquid nitrogen and stored at -80°C for subsequent determination of the pigment contents and RNA isolation.

### The qualitative analysis of pigments in chrysanthemum

Freeze dried tissue was used for the analysis. Samples of the ground freeze-dried petal tissue (50 mg DW) were initially extracted in 2 ml of petroleum ether and acetone (4:1), and 2 ml of methanol, acetic acid, water, trifluoroacetic acid (70:3:27:1) for 24 hours at 4°C in the dark with an absorption spectrum (220-700 nm; 400-500 nm) using a spectrophotometer (TU-1901; Beijing Puxi Co., Ltd).

### Gene isolation and bioinformatics analyses

 For reverse transcription polymerase chain reaction (RT-PCR) analysis, 3′-rapid amplification of the cDNA ends (RACE), and 5′-RACE, the total RNA was extracted from the five stages using TRIzol according to the manufacturer’s protocol. The first strand cDNA was synthesised using the reverse transcription (RT) system (Promega).

In a preliminary study, a cDNA library of *C. morifolium* was constructed (unpublished data). To isolate the 3′ and 5′ ends of the ABP genes, primers were designed and synthesised according to the ESTs of CmCHS, CmCHI, *CmF3H*, *CmF3*′H, CmDFR, *Cm3GT* and *CmANS*. Next, 3′ RACE and 5′-RACE were performed using the SMARTTM RACE cDNA Amplification Kit (Clontech). The final full-length sequences were then amplified using TransTaq DNA Polymerase High Fidelity (TransGen). The amplified product was then purified, ligated into the pGM-T vector, and cloned into the DH5α *Escherichia coli* strain followed by sequencing of all strands. BLAST analysis was performed to confirm the homologues to other plant anthocyanin biosynthesis genes. Sequence homology searches in Genbank were performed using the BLAST (http://www.ncbi.nlm.nih.gov) and DNAMAN programs. Structural analysis of the deduced protein was performed using the Expasy Molecular Biology Server (http://cn.expasy.org/tools/). Phylogenetic trees were constructed using the neighbour-joining method and the MEGA version 4.0 software.

### RT-PCR and qRT-PCR

Isolation of the total RNA from each stage of the 4 series and synthesis of the first strand cDNA were performed as previously described. The transcript levels of CmCHS, CmCHI, *CmF3H*, *CmF3*′H, CmDFR, *CmANS* and *Cm3GT* at the S0 to S4 stages were analysed using RT-PCR and quantitative real-time PCR according to the method described by Huang et al. [21]. The primers sequence used in this study were listed in Table S1 in File S1. *C. morifolium* β-actin (*CmActin*) was used as an internal control gene. The reactions were repeated three times.

### Transformation with *Agrobacterium tumefaciens*

Full-length *PCFH* cDNA was subcloned into the binary vector pBI121 in exchange for the *GUS* structural gene to construct vector 35S-PCFH. We selected *CmF3*′*H* as the targets for RNAi. Binary vectors with RNAi-induced inverted repeat structures were constructed as described by Nishihara et al. [22]. Approximately 500 bp of the gene was connected in sense and antisense into the pUC19 vector to construct the vector 35S-F3′Hir. The transverse thin cell layers (tTCLs) of the LPi were used as explants for the transformation experiments according to the methods described in our previous studies [23]. The *A. tumefaciens* strain EHA105, which contained either 35S-PCFH or 35S-F3′Hir, were used to inoculate the explants (the transgenic chrysanthemum were named pPCFH and pF3′Hir, respectively). For co-transformation of 35S-PCFH and 35S-F3′Hir, which is a mixed strain treatment, 35S-PCFH and 35S-F3′Hir, which contained a single T-DNA vector in two separate *Agrobacterium* strains, were transformed into the explants (the co-transformation of 35S-PCFH and 35S-F3'Hir were named pPCFH + pF3′Hir).

Bialaphos-resistant shoots were then transferred into the root-inducing medium. Plantlets were acclimatised and grown in a contained greenhouse. The flowers of the transgenic gentian plants were collected for further analysis and stored at -80°C until use.

### Flower colourimeter analysis, anthocyanin content measurement and HPLC analysis

The flower colour variables were measured on all of the positive lines of pPCFH, pF3′Hir and the co-transformation lines immediately after selection. The L^*^, a^*^, and b^*^ values were measured with a Konica Minolta CR-10 Chroma Meter (Minolta, Japan) on the opposite side of the medium part of each ligulate floret, all these ligulate florets were picked from the middle whorl of the capitulum[24]. The lightness coefficient ‘L^*^’, represents brightness and darkness, the ‘a^*^’ value represents greenish and redness as the value increases from negative to positive, and ‘b^*^’ represents bluish and yellowish. Anthocyanin compounds of pPCFH, pF3′Hir and the co-transformation lines were extracted from the petals using ethanol/water/acetic acid (10:9:1). The anthocyanin concentrations were determined by measuring the absorbance at 530 nm using a spectrophotometer and these experiments were repeated three times. For HPLC analysis, anthocyanins were analysed and characterised according the methods of Lai et al, 2007 [25], Lin and Harnly [26]. Malvidin-3,5-*O*-glucoside (Mv3G5G, Extrasynthese, France) was used as a standard for the quantitative analysis. All of the samples were analyzed in triplicate.

## Results

### Pigments in chrysanthemum

Flavonols/anthocyanins and carotenoids are often coexistence in the flower petals of chrysnthemums, and their combination increases colour cultivars. To avoid the obstruction of carotenoids on the flower phenotype during the GM experiment, we first carried out a qualitative analysis of pigments in the 4 chrysanthemum cultivars to find which cultivar could only accumulate anthocyanin in their petals. The pigment types in the 4 chrysanthemum cultivars is shown in Figure S1 in File S1. In LW, the main pigment was flavonols and carotenoids (with absorption peak at 342.8 nm / 468.00nm, 441.70nm, 417.00nm); in LR, the main pigment was flavonols, carotenoids and anthocyanins (with absorption peak at 334.5 nm, 529 nm indicated the existence of flavonols and anthocyanins, with absorption peaks at 468.00nm，441.70nm，419.00nm indicated the carotenoids); and LPi and LPu exhibited a similar pigment composition, which contained flavonols and anthocyanins (with absorption peak at 339.0nm/ 529.50nm and 340 nm/ 529.50 nm). The only type of anthocyanins in chrysanthemum was cyandin and the flavonols included apigenin, acacetin, eriodicyol, luteolin and diosmetin [25–27]. 

**Figure 2:**
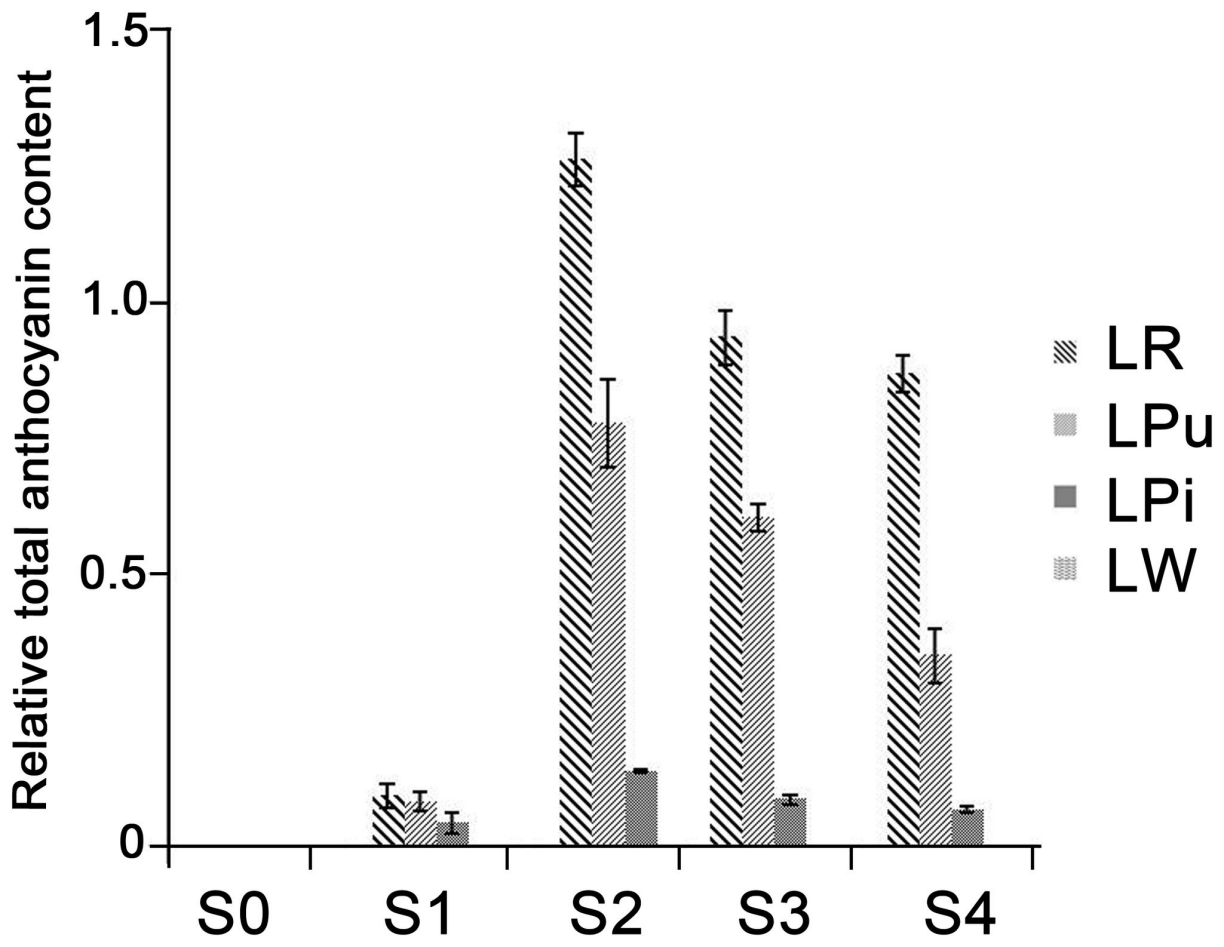
Anthocyanin accumulation in the petals of 4 chrysanthemum varieties at five developmental stages. Data shown are means of three biological replicates. Error bars denote standard error.

From stages S0 to S4, the anthocyanin contents in the 4 chrysanthemum varieties showed an initial increased in stage S2, and then a subsequent decreased. In S0, there was no anthocyanin accumulation in the flower petals (Figure 2).

### Sequence analysis of full-length cDNAs of CHS, CHI, F3H, F3′H, DFR, ANS and 3GT in chrysanthemum

Seven anthocyanin biosynthesis-related genes encoding predicted CHS, CHI, F3H, F3′H, DFR, ANS and 3GT were successfully isolated from the red ray floret of chrysanthemum encoding 397, 234, 356, 507, 373, 354, and 449 amino acid residues, respectively. The Genbank ID of these 7 genes were DQ521272, EU286277, DQ471438, KF313549, GU324979, EU810810 and JF433952, respectively. 

**Figure 3:**
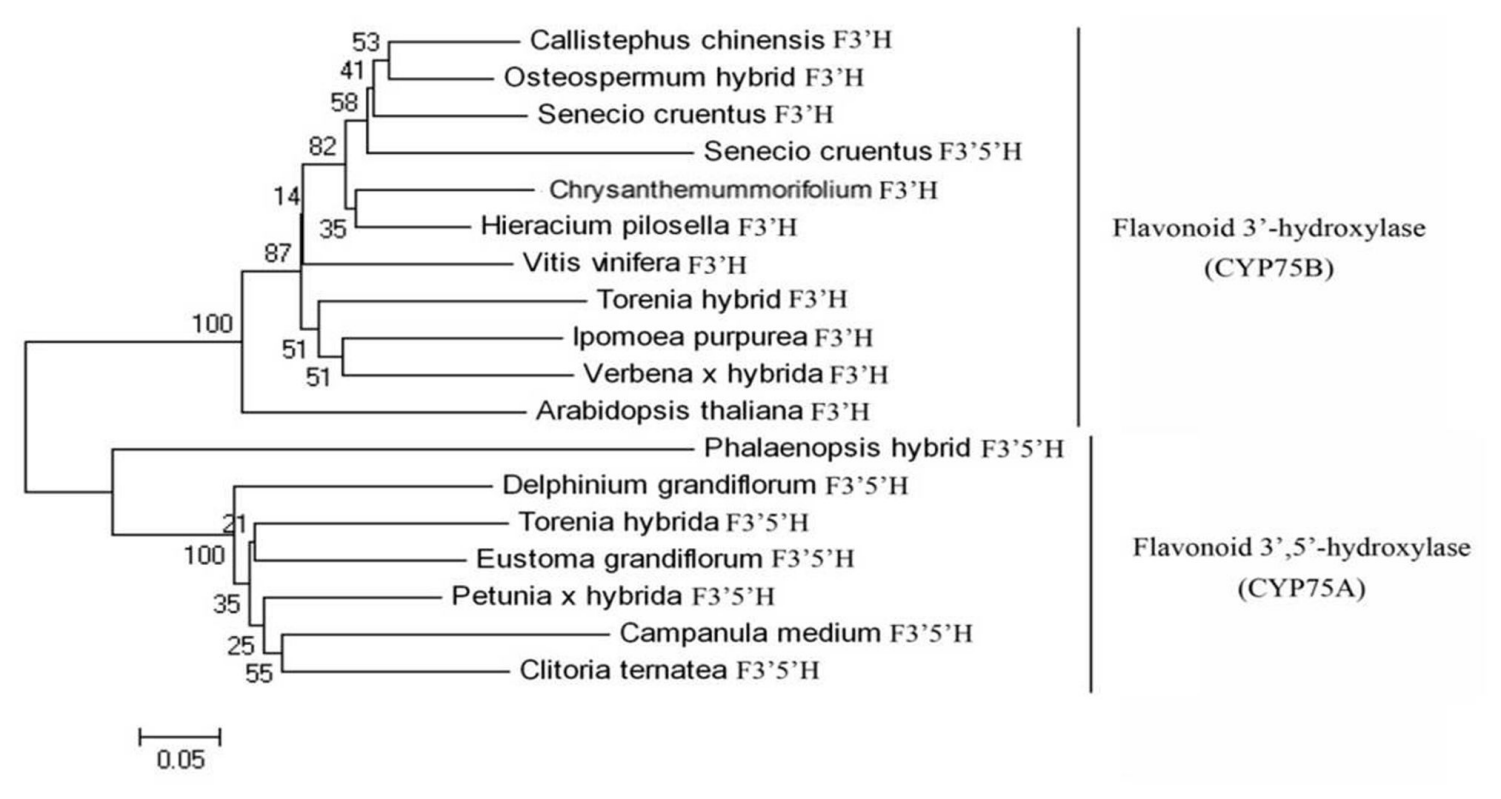
Phylogenetic analysis of F3′H and F3′5′H in various plant species calculated on the basis of the amino acid sequences. The numerals next to the branches indicate bootstrap values from 1000 replicates. The bar indicates an evolutionary distance of 0.05%. The accession numbers in the GenBank databases are as follows: flavonoid 3′-hydroxylase (F3′H): *Callistephus* *chinensis* (AAG49298), *Osteospermum* *hybrid* (ABB2989), *Chrysanthemum × morifolium* (ABY55858), *Senecio* *cruentus* (ABY55857), *Hieracium* *pilosella* (ABC47161), *Gerbera* *hybrid* (ABA64468), *Vitis* *vinifera* (BAE47006), *Torenia* *hybrid* (BAB87839), *Ipomoea* *purpurea* (AAR00229), *Verbena × hybrida* (BAE72874), *Arabidopsis* *thaliana* (NP 196416); flavonoid 3’, 5’-hydroxylase (F3’, 5’H): *Senecio* *cruentus* (AAX19888), Phalaenopsis hybrid (AAZ79451), *Delphinium* *grandiflorum* (AAX51796), *Torenia* *hybrida* (BAB20076), *Eustoma* *grandiflorum* (AAB17562), *Petunia × hybrida* (BAA03438), *Campanula* *medium* (O04773), *Clitoria* *ternatea* (BAE72870).

In CmCHS (Figure S2 in File S1), similar to other plants, the four conserved amino acid residues that are essential for the CHS active sites are Cys_167_, Phe_218_, His_306_, Asn_339_ [28]. Four strictly conserved residues (Thr_50_, Tyr_108_, Met_115_, Ser_192_) in CmCHI, which are important for catalytic activity of the CHI enzyme (Figure S3 in File S1) further support this gene identification[4] Importantly, the amino acid residues His_216_, Asp_218_ and His_291_ of CmF3H, which ligate ferrous iron, and Arg_301_ and Ser_303_, which participate in the 2-oxoglutarate binding (RXS motif), (Figure S4 in File S1) are the same at similar positions among plant F3Hs[29]. CmF3′H contained the conserved FxxGxRxCxG sequence (Figure S5 in File S1) in which the invariant cysteine residue serves as the fifth ligand to heme iron[30–32], and the phylogenic tree of F3′Hs and F3′5′Hs demonstrated that CmF3′H was grouped into the CYP75B subfamilies (Figure 3). Furthermore, phylogenetic analysis showed that F3′H and F3′5′H of Asteraceae formed the same cluster and F3′H was ancestral to F3′5′H (Figure 3), which was accordance with the report of Tanaka and Brugliera, 2013 [33]. Moreover, F3′5′H of Asteraceae was recruited from a pre-existing F3′H precursor gene and independently evolved from other F3′5′Hs[34] . Protein of the CmDFR gene exhibits a putative NADPH binding domain, which contains a specific conserved motif that was predicted to be related to substrate specificity and 5 strictly conserved amino acid residues in the DFR superfamily (Figure S6 in File S1)[35]. This is similar to other members of the 2OG-FeII_Oxy superfamily. Furthermore, CmANS contained the active sites of His_234_, His_280_ and Asp_236_ residues, which may coordinate iron at the catalytic centre of the iron-containing soluble oxygenases and 2-oxoglutarate-dependent enzymes . In addition, Arg_300_ and Ser_302_ residues were also conserved in CmANS, which may contribute to the specific binding of 2-oxoglutarate, and most likely provides a positive charge (Figure S7 in File S1)[36]. For Cm3GT, The deduced amino acid sequence showed a region between residues 403 and 446 (PSPG box, underlined in Figure S8 in File S1) that corresponds to the UDP-binding domain[37]. 

### The expression profiles of key structural genes

**Figure 4:**
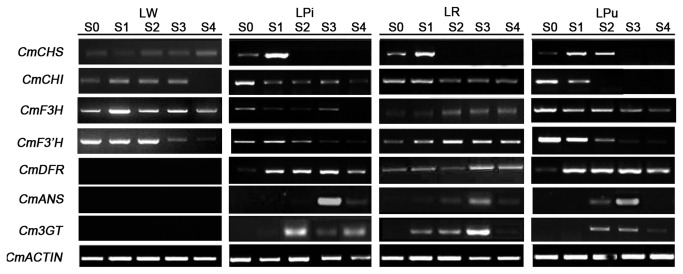
Expression pattern of key structural genes in chrysanthemum petals with different flower colour by RT-PCR.

**Figure 5:**
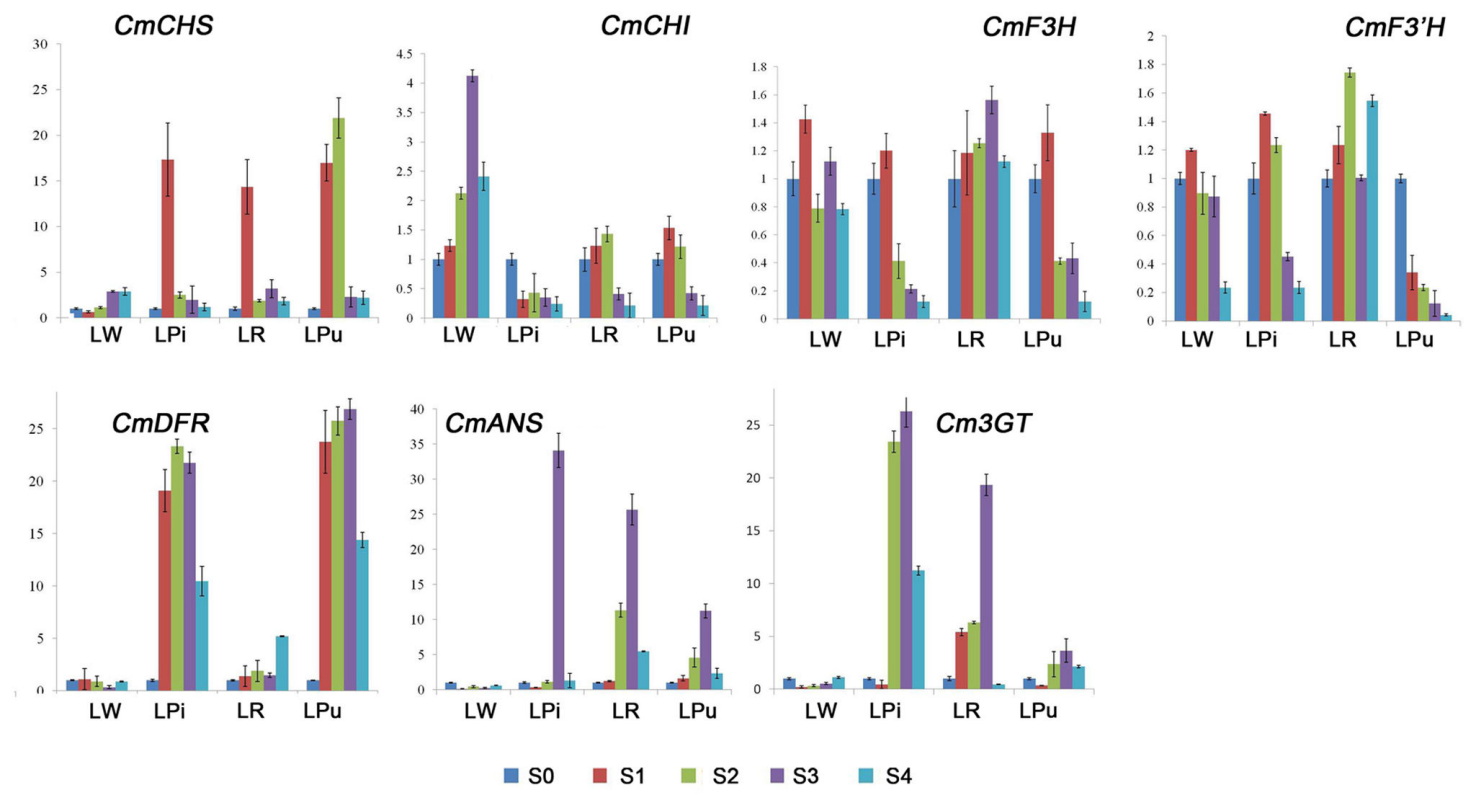
Expression pattern of key structural genes in chrysanthemum petals with different flower colour by qRT-PCR, Y-axis indicates the normalization fold expression of each gene. Data shown are means of three biological replicates, error bars denote standard error.

The expression profiles of the 7 structure genes, with the exception of *CmCHS* and *CmCHI*, were not detected in LW, which indicated the lack of ABP in white chrysanthemum cultivars. In LPi, LR and LPu, the expression of CmCHS, CmCHI, *CmF3H* and *CmF3*'*H*, which are regarded as upstream biosynthesis genes, were mainly expressed in stage S0 to S2, while the expression levels of downstream biosynthesis genes, which contained *CmDFR*, *CmANS* and *Cm3GT*, were continuously increased from S0 to stage S4 (Figure 4 and Figure 5). In addition, *CmF3*'*H* showed higher transcript levels in LR and LPu, but much lower levels in LPi, made it a good candidate receptor for GM. 

### Generation of transformed lines

**Figure 6:**
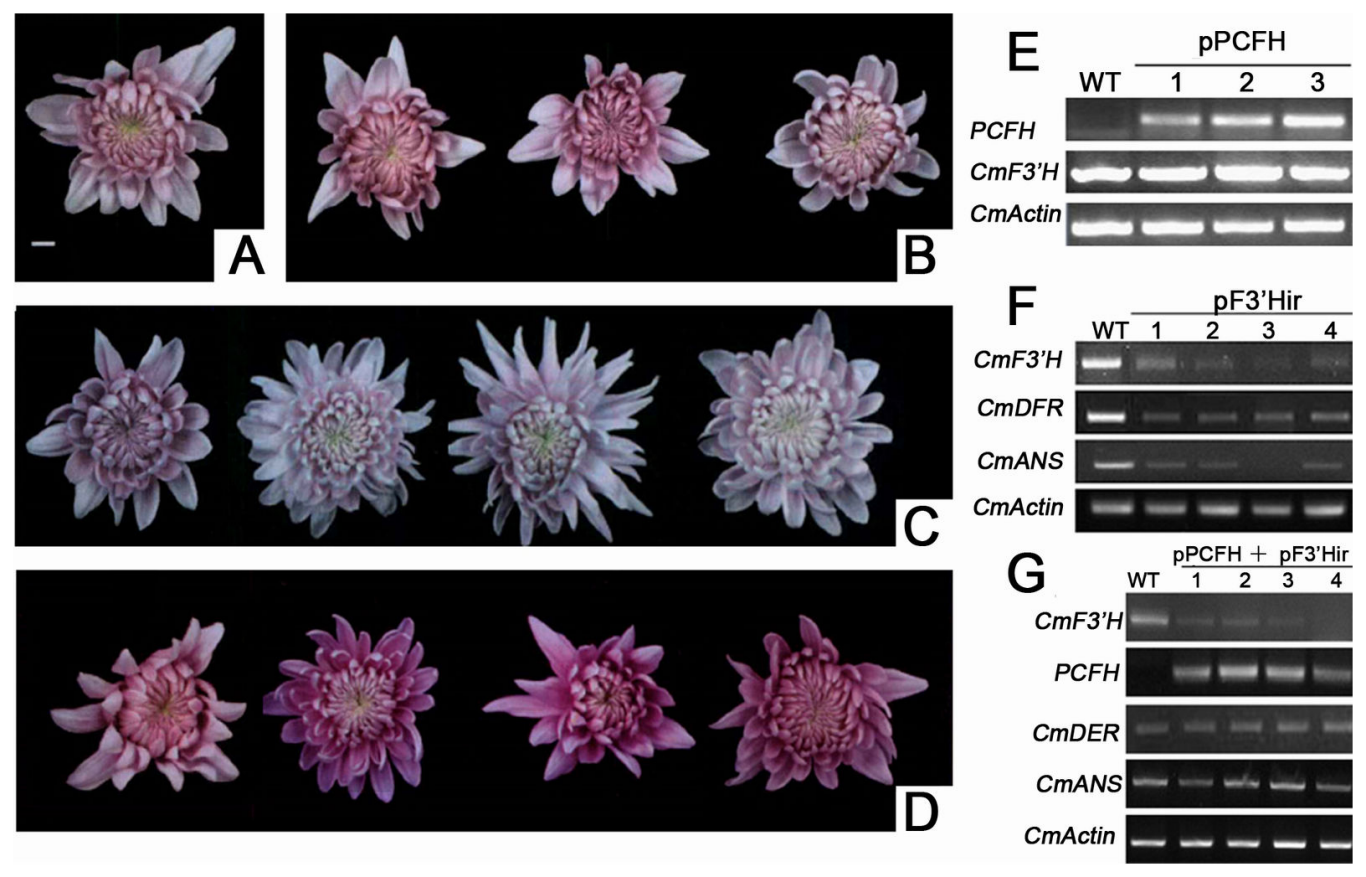
Typical flower phenotypes and RT-PCR of transgenic chrysanthemum plants. (A) Wildtype of LPi. (B) pPCFH lines 1, 2, and 3. (C) pF3′Hir lines1, 2, 3, and 4. (D) pPCFH +pF3′Hir lines1, 2, 3, and 4. (E) Expression analysis of *PCFH* and *CmF3*′*H* in the pPCFH lines. (F) Expression analysis of CmF3′*H*, *CmDFR* and *CmANS* in the pF3′Hir lines. (G) Expression analysis of *PCFH*, CmF3′*H*, *CmDFR* and *CmANS* in the pPCFH +pF3′Hir lines.

Overexpression of the *PCFH* transformants were produced from the 'LPi' cultivar using 35S-PCFH vector. Flowers from the transgenic lines did not show significant changes in colour compared with wildtype plants (Figure 6A and 6B). Moreover, the expression of *CmF3*′*H* in transgenic chrysanthemum plants also did not change (Figure 6E).

**Figure 7:**
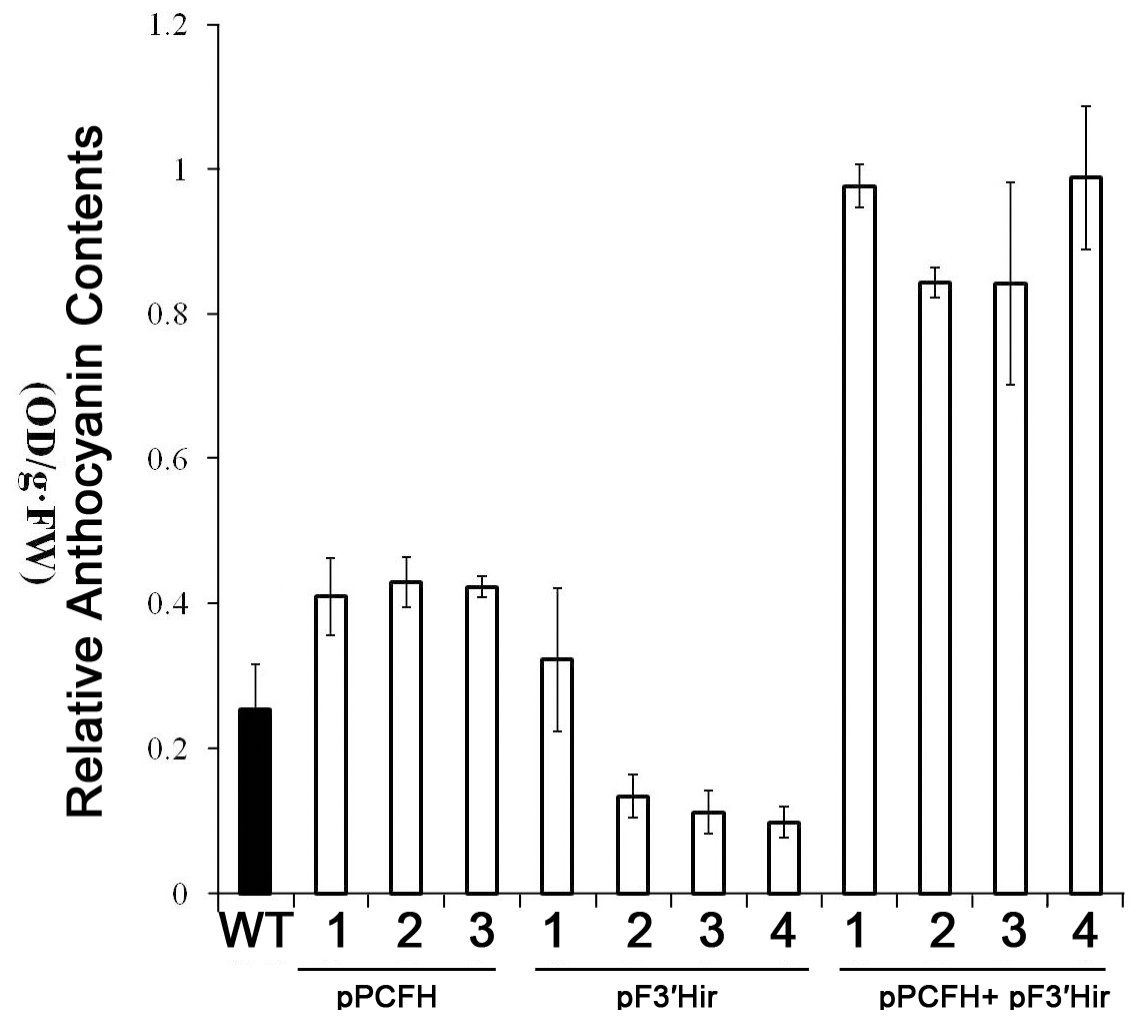
Anthocyanin accumulation in the ligulates of transgenic chrysanthemum flowers. The anthocyanin concentrations were determined spectrophotometrically by measuring the absorbance at 530 nm. Data shown are means of three biological replicates. Error bars denote standard error.

Suppression of CmF3'*H* transformants was performed in 'LPi' cultivars using 35S-F3′Hir vector. There was a marked reduction in the endogenous *CmF3*'*H* transcript in the positive transgenic lines (Figure 6C). In addition, anthocyanin accumulation in these transgenic flowers was significantly reduced by 20%-40% compared to wildtype plants (Figure 7). Furthermore, RT-PCR showed that the transcript levels of *CmF3*'*H* were significant decreased, and the transcriptional level of downstream genes were suppressed (Figure 6F)

**Figure 8:**
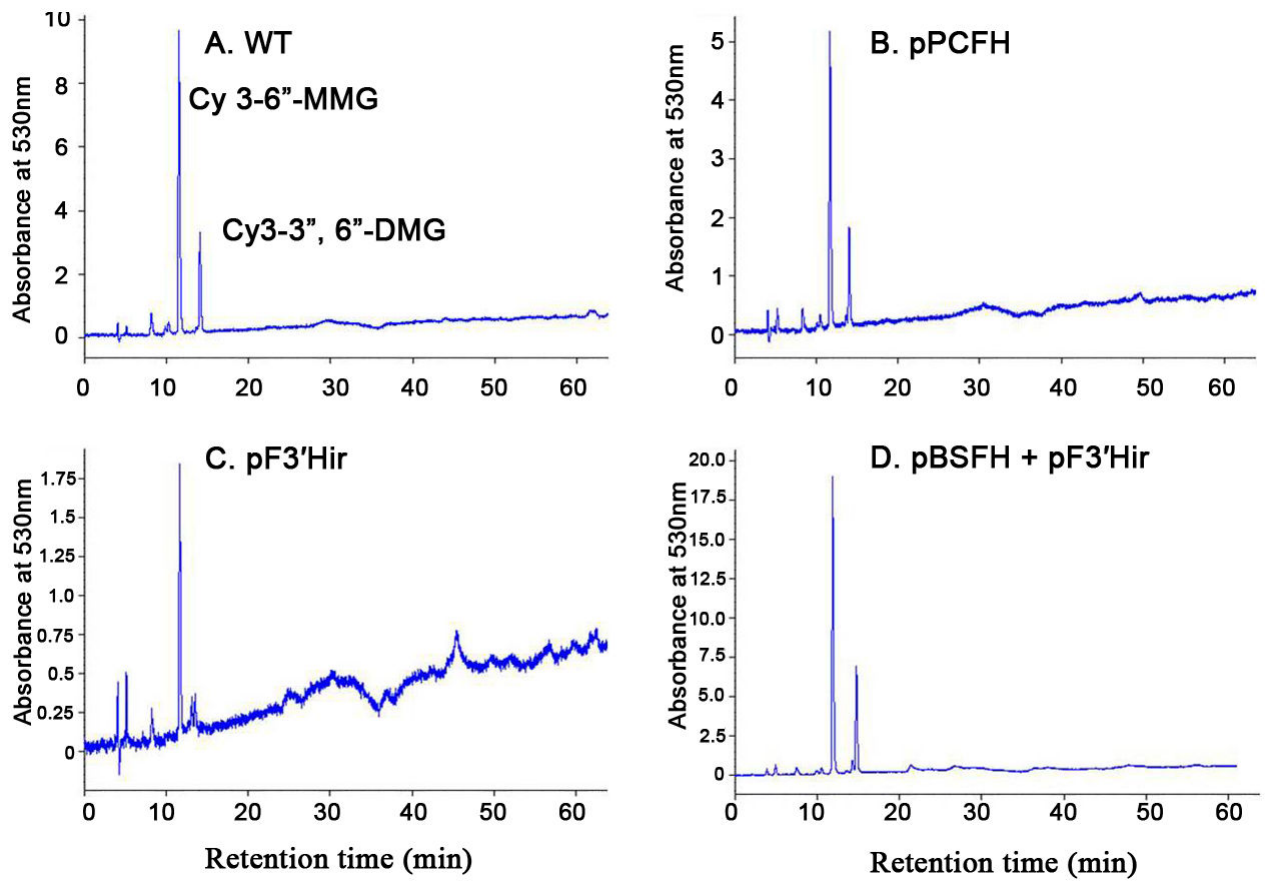
HPLC chromatograms for the ligulate extracts from selected transgenic lines and WT lines, Y-axis indicates the absorbance at 530 nm. Two peaks were Cy 3-6”-MMG and Cy3-3”, 6”-DMG respectively.

Because overexpression of *PCFH* did not produce significant phenotypic alterations in the colours of the chrysanthemum flowers, we performed a co-transformation of 35S-PCFH and 35S-F3′Hir. The flowers of the transgenic chrysanthemum plants exhibited a deeper colour compared to WT and pPCFH plant lines (Figure 6D). The change in colour, which was grossly observed by eye, was quantified by colour measurements using a colourimeter *CIEL* ^*^ *a* ^*^ *b* ^*^ (Table 1). We observed a significant decrease in b^*^ in the transgenic lines compared to wildtypes, suggesting that the colour became more blue; however, the HPLC and pigments measurement results demonstrated marked increased concentrations of anthocycanin (2.5-fold) (Figure 7 and Figure 8). However, the HPLC results showed that the co-transformation did not produce any new types of pigment. Compared with the pF3′Hir transgenic chrysanthemum plants, transcriptional level of endogenous *CmDFR* and *CmANS* gene recovered to the same intensity as wildtype plants (Figure 6G).

**Table 1:** Colour parameters (L^*^, a^*^, b^*^) of transgenic chrysanthemum.

	*L* ^*^	*a* ^*^	*b* ^*^
WT	70.22±1.14	4.84±0.11	1.51±0.03
pPCFH -1	59.76±1.67	11.23±0.20	1.53±0.01
pPCFH -2	58.25±0.29	13.79±0.09	1.47±0.02
pPCFH -3	62.41±1.22	13.11±1.05	1.35±0.02
pF3′Hir *-1*	85.25±0.18	2.42±0.03	6.21±0.48
pF3′Hir -2	80.14±0.76	3.61±0.06	6.18±0.43
pF3′Hir -3	80.64±1.24	3.99±0.11	7.23±0.64
pF3′Hir -4	82.16±1.25	4.07±0.04	5.48±0.35
pPCFH+pF3′Hir *-1*	19.73±0.25	22.32±1.56	-10.12±0.23
pPCFH+pF3′Hir *-2*	15.24±0.93	24.61±0.27	-11.45±0.04
pPCFH+pF3′Hir *-3*	16.19±0.38	25.79±0.87	-10.27±0.12
pPCFH+pF3′Hir *-4*	14.36±1.15	24.85±0.46	-9.88±0.74

For each cultivar, the means within a column are followed by different letters, which are significantly different at p<0.05.

## Discussion

### Analysis of the key structural gene expression patterns in different colour chrysanthemum ray florets

During the past few decades, nearly all of the enzymes involved in ABP for different flower phenotypes have been determined. The lack of activity in one or more genes of these pathways may inhibit the synthesis of anthocyanins, which results in the inability to form colourful anthocyanins[38]. In the present study, we found that the expression of genes encoding enzymes of anthocyanin biosynthesis is similarly regulated in ray florets among the 4 chrysanthemum cultivars with different colours. However, in chrysanthemum ray florets, the red colour has also been contributed for carotenoids, or combined with red or magenta anthocyanins [8], which was according to our study, the pigments of LPi and LPu were cyanidian. 

Analysis of the expression patterns of key structural genes suggest that anthocyanin biosynthesis-related genes in chrysanthemum may be classified into two groups on the basis of their temporal expression patterns during ray floret development. The first group consists of CmCHS, CmCHI and *CmF3*’*H*, which are more highly expressed at early development stages compared to late stages. These findings indicated that a sufficient number of precursors were needed to accumulate for anthocyanin and other co-pigment biosynthesis during the early stages of inflorescence growth. The second group consists of *CmF3H*, *CmDFR* and *CmANS*, which are necessary for anthocyanin biosynthesis and are coordinately expressed throughout all of the stages of ray floret development. In addition, the gene expression profile of these four genes correlates with pigment accumulation. Similar results have also been reported in apple[39], litchi[40] and *Pyrus pyrifolia*[41], indicating that multiple late genes determine the anthocyanin accumulation among the different genotypes.

The expression of CmCHS, CmCHI, *CmF3H*, *CmF3*′H, CmDFR and *CmANS* in chrysanthemum showed that the genes responsible for anthocyanin synthesis accumulated during the early stages of capitulum development (Figure 4 and Figure 5), which provided a reference for the selection of a suitable developmental stage for transgenic studies in chrysanthemum. DFR is one of the most important key enzymes for anthocyanin biosynthesis, in petunia(*Petunia hybrida*), the PhDFR proteins do not accept monohydroxylated DHK, and thus, cannot produce the corresponding monohydroxylated pelargonidin anthocyanins [42]. F3′H catalyses the hydroxylation of flavanones, flavones and flavonols. It often competes against the introduced F3′5′H. To avoid these competitions, it is necessary to downregulate F3′H and DFR [43]. However, because the only ABP in chrysanthemum is the cyanidin-based pathway, there is no DFR competition with F3′H. Thus, regulation of *CmF3*′*H* appeared to be important in the production of blue flower pigmentation in chrysanthemum. 

On the basis of the above analysis, we concluded that the most effective strategy to obtain the blue flowers by genetic modification was to suppress the activity of endogenous enzymes which produce cyanidin, and to introduce an appropriate exogenous *F3*′*5*′*H* to produce delphinidin.

### Key roles of CmF3'H in chrysanthemum

It is feasible to generate white, yellow, red and blue flowers by engineering the pathway; both by overexpression of heterogenous genes and/or downregulation of endogenous genes [11]. A growing number of potentially useful molecular tools to engineer blue or red flower colours have now become available. However, only modification of B-ring hydroxylation has been effectively demonstrated in modifying flower colour[13]. The F3′H and F3′5′H enzymes play important roles in flower colour by determining the B-ring hydroxylation pattern of anthocyanins. In genetic breedings of the blue rose, cultivars with no F3′H activity were selected to enhance the blue hue of the transgenic petals and to achieve a high content of delphinidin [12]. In the present study, the transcriptional levels of *F3*'*H* in LPi were much lower compared to other cultivars, which was a good candidate explant to create the blue chrysanthemum. Suppression of *CmF3*'*H* successfully changed the anthocyanin profiles and flower colour in chrysanthemum. A shift from predominantly cyanidin-derived pigments to non-pigment transgenic lines was achieved and revealed a concomitant shift in the CIEL^*^a^*^b^*^ value. Previous reports have demonstrated that GM toward *F3*'*H* in other ornamental plants has successfully generated new colour flower cultivars. In *Osteospermum*, suppression of *F3*'*H* by RNAi and the introduction of gerbera DFR resulted in pelargonidin accumulation [44]. Furthermore, downregulation of *F3*'*H* and *flavonol synthase* (*FLS*) genes as well as the overexpression of the gerbera *DFR* gene in tobacco resulted in pelargonidin production[45]. Moreover, heterogenous expression of Gentian *F3*'*H* modulated the intensity of flower pigmentation in transgenic tobacco plants [46] .

As shown in pF3′Hir transgenic chrysanthemum plants, we observed the down-regulated expression of *CmANS* and *CmDFR*, which was important to produced the cyanidins. The same phenomenon was also observed in *I. quamoclit*, where *F3*'*H* gene expression was dramatically reduced in *I. quamoclit* and DFR was unable to catalyse dihydroquercetin, a precursor of cyanidin [47]. In addition, expression of the introduced *F3*′*H* resulted in the suppression of the endogenous *ANS* gene and production of peonidin and quercetin in transgenic flowers, thereby resulting in pink flowers in the petunia [48] .

### Overexpression of an exogenous F3'5' H failed to create blue but produced brighter red chrysanthemum lines

Loss-of-function or reduced expression of the gene encoding the branching enzyme F3'5'H was the main reason for the loss of flux down the delphinium branch of the ABP. Specific studies demonstrated an accumulation of delphinidin-derived anthocyanins by overexpression the *F3*'*5*'*H* have been reported in the carnation[49] and rose[12], while inhibition of both the *F3*'*H* and the *F3*'*5*'*H* genes modifies the colour and promotes cyanidin- and pelargonidin-based pigment accumulation in flowers of *Torenia* [50], *Nierembergia* [51] and *Osteospermum* [44]. 

Delphinidin-based pigments were not detected in the flowers of the transgenic chrysanthemum using HPLC, anthocyanin content measurements and CIEL^*^a^*^b^*^ analysis. Co-expression of pPCFH and pF3′Hir could not successfully create blue chrysanthemums; however, the cyandin content increased 2-fold in the transgenic lines compared to WT. These results indicated that overexpression of a gene resulting in a target compound was not sufficient and that it was necessary to downregulate competing pathways to accumulate a blue compound [6].

 In our study, overexpression of exogenous *F3*'*5*'*H* results in no phenotypic change in chrysanthemum, one explanation for this absence may be due to the competition of endogenous CmF3'H with PCFH. A second reason may be that some chrysanthemum family species, which produce delphinidin-based anthocyanins, contain a CYP75B- and not a CYP75A-type F3'5'H, indicating that the chrysanthemum family may have lost the F3'5'H function during their evolution. F3'5'H function was then reacquired by gene duplication and neo-functionalisation of the CYP75B-type gene[13]. However, our previous studies showed that overexpression of *PCFH* could produce delphinidin in tobacco, and the transgenic lines showed a blue flower phenotype [20]. Expression of the *Campanula medium F3*'*5*'*H* gene resulted in a more efficient accumulation of delphinidin (up to 99%) compared with the petunia or Lisianthus *F3*'*5*'*H* gene in tobacco, which indicated that the gene origin is important for successful engineering [52]. and which was accordance with the report of Gion et al., 2012 [53] that in chrysanthemum, expression of a pansy *F3*'*5*'*H* gene under the control of rose chalcone synthase promoter resulted in transgenic chrysanthemum with flower colour change from pink to violet and accumulation of delphinidin.

Similar challenges are also posed in the breedings of roses with blue flowers. The expression of *F3*'*5*'*H* genes from the petunia, gentian or butterfly pea in rose resulted in no or little delphinidin accumulation in the petals of the transgenic plants, although these genes are functional in the petunia, carnation or yeast. In contrast, the expression of the pansy (*Viola spp* ) *F3*'*5*'*H* gene resulted in a significant amount of delphinidin-derived anthocyanins accumulating in the petals of transgenic rose plants [54]. Substrate specificity is an important consideration in delphinidin production. Dihydroflavonols are reduced in corresponding 3,4-cisleucoanthocyanidins by the activity of DFR. In some plant species, such as petunia (*Petunia hybrida*) and *Cymbidium*, DFR demonstrates strict substrate specificity and cannot utilise dihydrokaempferol. Thus, these species lack pelargonidin-based anthocyanins and have no flowers of an orange/brick red colour [1] . According to our study, there was no pelargonidin in chrysanthemum, and the 134-136 AA in CmDFR was Asn, which could only utilise DHQ [55]. Our group has isolated five *DFRs* from cineraria, among which two utilises DHM to produce delphinidin. Thus, our strategy to produce blue chrysanthemums will focus on following two points: (1) selection of a more effective *F3*'*5*'*H*. *F3*'*5*'*H* from other species should be transformed into the *F3*'*Hi* lines to produced DHMs; and (2) suppression of the inner *DFR* in chrysanthemum and introduction of *ScDFR* to produce delphinidin in chrysanthemum.

## Supporting Information

File S1 **Supporting information file containing the following files.** **Table S1**, primer sequences used in this study. **Figure S1**, UV/Vis spectra of pigments and pigment types in different colour series of chrysanthemum ray florets. (A:absorption spectrum at 220-700nm; B:400-500 nm) **Figure S2**, Amino acid sequence alignment of CmCHS from different species; the asterisks indicate the conserved amino acid residues or active sites. **Figure S3**, Amino acid sequence alignment of CmCHI from different species; the asterisks indicate the conserved amino acid residues or active sites. **Figure S4**, Amino acid sequence alignment of CmF3H from different species; the asterisks indicate the conserved amino acid residues or active sites. **Figure S5**, Amino acid sequence alignment of CmF3'H from different species; the asterisks indicate the conserved amino acid residues or active sites. **Figure S6**, Amino acid sequence alignment of CmDFR from different species; ▼shows the conserved amino acid residues in the DFRs, the box is a putative NADPH binding region. **Figure S7**, Amino acid sequence alignment of CmANS from different species, ▼shows the conserved amino acid residues in the ANSs. **Figure S8**, Amino acid sequence alignment of Cm3GT from different species, underline shows the conserved region (PSPG motif) in the 3GTs.
(RAR)
